# A Comparative Evaluation of the Quality and Feasibility of ‘Over-the-Head’ Cardiopulmonary Resuscitation by a Single Rescuer: Pocket Mask vs. Bag-Valve Mask—A Pilot Study

**DOI:** 10.3390/healthcare13121428

**Published:** 2025-06-14

**Authors:** Silvia San Román-Mata, Marc Darné, Ernesto Herrera-Pedroviejo, Martín Otero-Agra, Rubén Navarro-Patón, Roberto Barcala-Furelos, Silvia Aranda-García

**Affiliations:** 1 REMOSS Research Group, Faculty of Education and Sport Sciences, University of Vigo, 36005 Pontevedra, Spain; silviasanroman@ugr.es (S.S.R.-M.); martinoteroagra@gmail.com (M.O.-A.); 2 Department of Nursing, University of Granada, 18071 Granada, Spain; 3 GRAFAIS Research Group, Institut Nacional d’Educació Física de Catalunya (INEFC), University of Barcelona, 08007 Barcelona, Spain; marcdarne123@outlook.com (M.D.); saranda@gencat.cat (S.A.-G.); 4 Blanquerna Facultad de Ciencias de la Salud, Universitat Ramon Llull, 08025 Barcelona, Spain; ernestohp1@blanquerna.url.edu; 5 School of Nursing, University of Vigo, 36005 Pontevedra, Spain; 6 Faculty of Teacher Training, Universidade de Santiago de Compostela, 27001 Lugo, Spain; ruben.navarro.paton@usc.es

**Keywords:** basic life support training, resuscitation quality, ventilations, mouth-to-pocket-mask, training

## Abstract

**Aim**: The present study evaluated the feasibility and quality of cardiopulmonary resuscitation (CPR) performed by a single rescuer, comparing the over-the-head (OTH) technique using mouth-to-pocket mask ventilation with bag-valve mask (BVM) ventilation. The study analyzed the chest compression (CC) quality, ventilation adequacy, interruption minimization, and the rescuers’ perceived difficulty. **Methods**: A randomized simulation crossover study was conducted with 26 lifeguard students trained in basic life support and both ventilation techniques. All of the participants performed two solo CPR trials (2 min each) using OTH with a pocket mask or BVM on a manikin connected to a feedback system (Little Anne QCPR, Laerdal). The overall CPR quality, ventilation, and CC quality were assessed, along with the perceived difficulty (scale 0–5). A 5 min rest was provided between the trials. **Results**: The overall CPR quality was excellent for both techniques with a median of 98% (IQR: 97–99) for BVM-OTH and 99% (IQR: 94–99) for Pocket-OTH (*p* = 0.31). The ventilation quality was better when using BVM-OTH (100%, IQR: 99–100) compared to that with Pocket-OTH (99%, IQR: 77–100; *p* = 0.046). No differences were found in the CC quality (99%, IQR: 99–100; *p* = 0.24). However, Pocket-OTH had more CCs and shorter interruption times (*p* ≤ 0.001). The perceived difficulty was low for both techniques. **Conclusions**: Both techniques enable high-quality CPR when performed alone. Given that no clinically relevant differences emerged in the resuscitation quality, the OTH technique using a pocket mask offers a viable alternative, particularly in scenarios with a single rescuer and limited resources.

## 1. Introduction

Cardiopulmonary resuscitation (CPR) is an intervention performed in emergency situations and can mean the difference between life and death in cases of cardiac arrest [1]. When a person is unresponsive and not breathing normally, immediate CPR is crucial to improve their chances of survival [2,3]. However, the circumstances surrounding a cardiac arrest can vary significantly, and the approach must be adapted to the underlying etiology [4,5]. In the case of cardiac arrest due to drowning, defined as the process of experiencing respiratory difficulties due to submersion or immersion in a liquid [6], reversing hypoxia by trained first responders becomes especially important, making effective ventilation a key component of resuscitation [7,8,9]. When approaching a cardiac arrest due to drowning, ventilations for reoxygenation play a critical role [10]. According to the recommendations of the European Resuscitation Council Guidelines (ERCGR, 2021) [11], trained first responders should start CPR with five rescue breaths, followed by cycles that alternate between 30 chest compressions and 2 ventilations.

In ideal conditions, CPR should be performed by multiple trained rescuers equipped with tools that facilitate oxygenation, such as the bag-valve mask (BVM) device. However, in aquatic environments such as pools or beaches, it is not uncommon for a single rescuer to handle all aspects of the drowning chain of survival, from prevention to critical interventions like CPR [12]. Among the mechanisms that trained first responders can use for ventilation, the pocket mask, similarly to a BVM, is another common device [13] that is easy to carry in different scenarios involving an aquatic incident response [14].

In cases in which a rescuer has a BVM or pocket mask, the ‘over-the-head’ (OTH) resuscitation technique offers an alternative in which ventilations and compressions are performed at the head of the patient [15,16]. This technique allows a single rescuer to perform chest compressions and ventilations effectively, even in confined spaces or when it is not possible to position oneself beside the victim [15,17]. The OTH technique emerged two decades ago with the aim of addressing the knowledge gap regarding effective resuscitation under special circumstances (e.g., confined spaces such as airplane aisles). Nonetheless, in recent years, it has regained the interest of the scientific community, especially when ventilation plays a significant role [14].

Previous studies have explored the efficacy of the over-the-head (OTH) CPR technique. Perkins et al. [15] compared OTH with standard CPR and concluded that OTH is a viable alternative in confined spaces, given that it offers comparable effectiveness and some advantages in terms of hand placement. Similarly, Ćwiertnia et al. [16] assessed the CPR quality of both standard and OTH positions when performed by a single paramedic and found that the OTH method may enhance certain CPR quality parameters. However, these studies did not specifically focus on scenarios involving drowning victims or compare different ventilation methods within the OTH technique. The present study aims to fill this gap by evaluating and comparing the feasibility and effectiveness of CPR performed by a single rescuer using the OTH technique with either a pocket mask or a bag-valve mask (BVM) in a simulated drowning context.

The aim of the present research was to compare the feasibility and effectiveness of CPR using the OTH technique with mouth-to-pocket-mask and BVM ventilation when performed by a single rescuer. The present study will analyze the quality of chest compressions, adequacy of ventilation, and minimization of interruptions to the compression–ventilation cycle, as well as the rescuers’ perceived difficulty of execution.

## 2. Materials and Methods

### 2.1. Study Design

**Figure 1:**
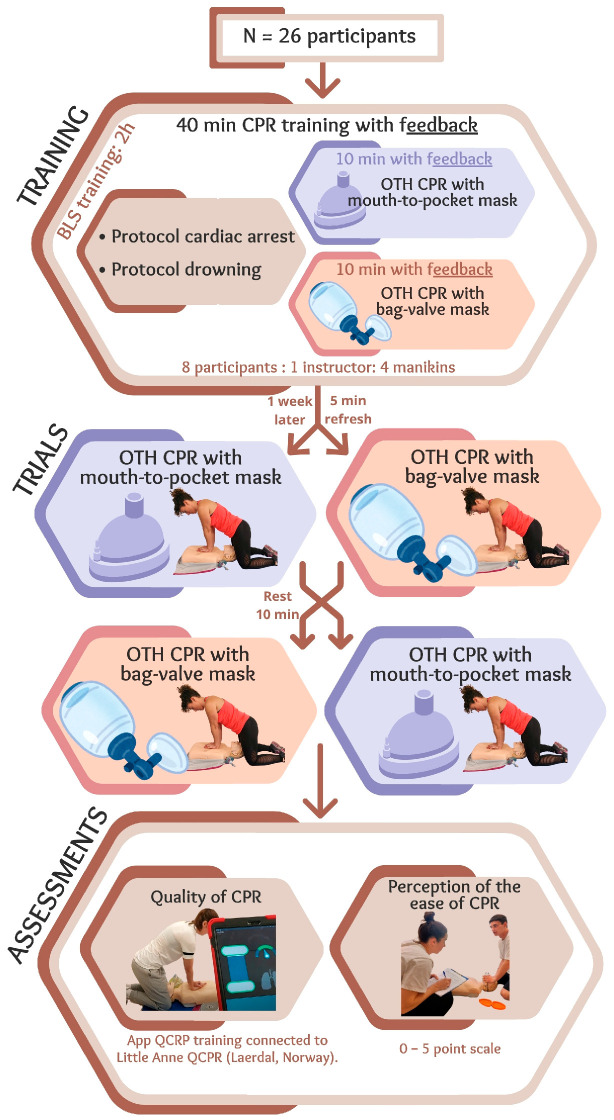
Flow chart and study design. OTH: over-the-head, CPR: cardiopulmonary resuscitation.

A randomized simulation crossover design was used to analyze differences between BVM and pocket mask use when performing the OTH technique during a two-minute CPR test. This crossover design was appropriate as it minimized inter-individual variability by using each participant as their own control. The order in which tests were performed were randomized with a ten-minute rest period between tests to minimize the effect of fatigue (Figure 1). The study was conducted in accordance with the principles of the Declaration of Helsinki. It was approved by the Ethics Committee of Faculty of Education and Sport Sciences of the University of Vigo with the reference number 13-050724.

### 2.2. Participants

A non-probabilistic convenience sampling approach was employed for the present study due to the availability of participants. Participants were recruited from students enrolled on the Bachelor’s Degree in Physical Activity and Sports Sciences, who are all required to be trainee lifeguards. A convenience sample of 26 participants was recruited. No formal sample size calculation was performed, as this was a pilot study. Participants were excluded if they reported having any impediment to performing CPR or failed to reach a 70% CPR quality after completing a CPR training course. Signed informed consent was obtained from all participants prior to the study starting.

### 2.3. CPR Training Course and OTH Refresher for a Single Rescuer

All participants in the present study underwent intensive two-hour basic life support training, which included predominantly practical content on the basic life support protocol and drowning victim management [8,11,18], including standard CPR techniques and the OTH technique for a single rescuer, with two ventilation methods: mouth-to-pocket mask (Pocket mask, Laerdal, Stavanger, Norway) and BVM (Adult Bag II Resuscitation, Laerdal, Norway) (Figure 1). For 40 min, participants practiced CPR whilst receiving constant feedback from the QCPR training app (Laerdal, Norway) connected to the training manikin (Little Anne QCPR, Laerdal, Norway). This app provides real-time feedback through a visual system that monitors parameters related to the quality of chest compressions and ventilations. The Laerdal QCPR App generates an overall CPR quality percentage based on compliance with key performance indicators from international guidelines, although its exact algorithm is not publicly disclosed. These parameters adhered to current international recommendations which determine that high-quality CPR includes chest compressions performed at a rate of 100–120 per minute to a depth of 5–6 cm, whilst achieving full chest recoil and minimal interruptions. Further, ventilations should be sufficient to produce visible chest rise [18], typically between 400 and 600 mL of air. Of these 40 min, 20 min were spent being trained in resuscitation with a single rescuer positioned OTH, 10 min were spent ventilating with a mouth-to-pocket mask, and 10 min were spent ventilating with a BVM. Training was conducted in groups of eight students per certified instructor, with four training manikins per group. At the end of the training, participants were required to achieve an OTH CPR quality above 70% with both ventilation methods, as established by quality standards [19]. This threshold, originally proposed by Perkins et al. in 2004 [19], is widely accepted in resuscitation science as the minimum level of performance from which effective tissue oxygenation can be expected in real-life scenarios.

On week later, and prior to starting the trials, all participants performed a brief five-minute instructor-guided roller refresher on both OTH techniques when applied by a single rescuer (BVM and pocket mask ventilation). The same feedback system to that used during training was again used here.

### 2.4. Clinical Scenario for Simulation with OTH Resuscitation: Mouth-to-Pocket Mask vs. BVM

The following scenario was presented to all participants for each of the trials:

‘This adult victim has just been rescued from the pool. They are not breathing and are in cardiac arrest. You must resuscitate them while positioned OTH. You have this equipment available for ventilation’ (BVM or pocket mask, depending on randomization).

**Figure 2:**
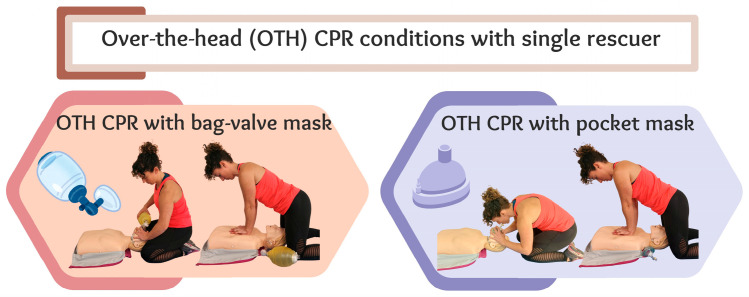
Over-the-head (OTH) cardiopulmonary resuscitation (CPR). Ventilation techniques: mouth-to-pocket mask and bag-valve mask.

Each participant completed two trials with two different ventilation conditions, i.e., with BVM and with mouth-to-pocket mask (Figure 2). Each trial consisted of performing five initial ventilations, as stipulated by the drowning protocol [8], followed by two minutes of resuscitation (30 chest compressions and 2 ventilations) without a feedback system. Both trials were OTH CPR performed by a single rescuer. A 30 min rest was scheduled between the two tests to prevent fatigue effects. The order of the conditions was randomized using a computer-generated list, which assigned participants to one of the two possible sequences according to their order of inclusion. The allocation was concealed from both participants and investigators until each test.

### 2.5. Assessment and Variables

Variables related to the following were recorded: (a) sociodemographic variables; (b) CPR performance; and (c) perception of ease/difficulty in performing CPR.

(a) Sociodemographic variables: Demographic data were recorded, including age (years), weight (kg), height (m), and sex.
(b) CPR performance: Data were collected on CPR quality (%), number of effective initial ventilations (#), ventilation quality (%), effective ventilations, quality of chest compressions (CCs) (%), number of CCs, CC fraction (%), average CC rate (CC/min), CCs with correct rate (%), CCs with correct depth (%), and CCs with correct recoil (%). These data were recorded using the QCPR training App (Laerdal, Norway) connected to the manikin (Little Anne QCPR, Laerdal, Norway). The study variables calculated by default by this app, which are based on international quality standards, are chest compressions at a depth of 5–6 cm and a rate of 100 to 120 compressions per minute, and ventilations delivering between 400 and 600 mL of air [20].
(c) Perception of ease/difficulty when performing CPR: Participants were asked how easy it was to perform the resuscitation techniques for both conditions in terms of overall CPR, CC, and ventilation. Responses were provided along a scale that ranged from 0 to 5 (0: very easy; 1: easy; 2: somewhat easy; 3: somewhat difficult; 4: difficult; 5: very difficult).

### 2.6. Statistical Analysis

Data analysis was conducted using IBM SPSS Statistics software version 20 (IBM Corp., Armonk, NY, USA). Qualitative variables were described using relative and absolute frequencies. Quantitative variables were described using measures of central tendency (median) and measures of dispersion (interquartile range). For comparisons between the different study trials, the paired Student’s *t*-test was used for parametric variables, whilst the Wilcoxon rank-sum test was used for non-parametric variables. Normality was tested using the Shapiro–Wilk test. A significance level of *p* < 0.05 was established for all analyses.

## 3. Results

### 3.1. Sociodemographic Results

The final sample included 26 participants, of which 17 were men and 9 were women, with a median age of 20 years (IQR: 20–23), weight of 70 kg (IQR: 63–74), and height of 1.74 m (IQR: 1.68–1.80).

### 3.2. CPR Performance

The outcomes pertaining to the comparative analysis of CPR when conducted with two different ventilation conditions (BVM-OTH vs. Pocket Mask-OTH) are presented in Table 1. Outcomes are presented for the overall CPR quality, ventilation quality, CC quality, and perceived difficulty in performing CPR. The outcomes indicate excellent performance for both conditions across all of the analyzed variables. Statistically significant differences were found for three variables: the ventilation quality, number of CCs, and CC fraction. No significant differences were found in the CPR performance based on the order in which the tests were performed.

### 3.3. Perception of Ease/Difficulty When Performing CPR

Finally, the study participants reported generally perceiving BVM and pocket mask use to be easy for performing CPR and CC, as well as somewhat easy for performing ventilations, with no statistically significant differences emerging for any comparison (Table 2).

## 4. Discussion

The aim of the present study was to evaluate the feasibility of CPR when performed by a single trained first responder using a pocket mask when compared with the use of a BVM. The study also aimed to assess the perceived difficulty of using each technique. In this way, the study sought to address a knowledge gap in pocket mask use for over-the-head CPR, as it is a common resource and an accessible resuscitation technique available to rescuers and lifeguards.

The main findings are as follows: (a) the use of a pocket mask by a single first responder when performing the OTH technique is feasible and does not pose any handicap compared to the use of a BVM; (b) study participants considered pocket masks to be easy or somewhat easy to use, which demonstrates that they do not represent a technical barrier to CPR when performed by a single rescuer.

Based on present findings, it can be concluded that it is feasible for a single rescuer to perform CPR using either a pocket mask or a BVM. The pocket mask has previously been shown to be an effective method for ensuring adequate tidal volume during resuscitation by a trained non-medical responder [20]. While the present study found some significant differences in ventilations favoring the BVM, these differences lack clinical relevance (e.g., only a 1% difference in ventilation quality) and the overall performance was excellent in both cases (median of 100% with BVM and 99% with pocket mask). Such a small difference would not be expected to affect the effectiveness of resuscitation in a real-life scenario. The participants demonstrated good proficiency with both devices, which likely contributed to their perception that both ventilation devices are easy to use.

In the present study, both the chest compression fraction and the number of chest compressions were slightly higher in the pocket mask condition compared to the BVM condition. This difference may be explained by the time required to handle the equipment during each ventilation cycle. In the case of the pocket mask, the mask was already placed on the victim’s face and held in position with an elastic strap, allowing the participants to ventilate quickly and without the need for repositioning. In contrast, when using the BVM, the participants had to pick up the device from the floor and create a manual seal before ventilating, which required more time and slightly reduced the duration available for chest compressions. Previous research has shown that the time required to establish an effective seal when using a BVM can affect the CPR efficiency, particularly in terms of minimizing interruptions to compressions [21]. This operational difference, although small, may have implications for training and protocol development, especially in settings where only one rescuer is present.

A novel aspect of the present study was its assessment of the pocket mask when used by a single rescuer performing the ‘over-the-head’ CPR technique. Previous scientific literature on lifeguards using the standard CPR technique has revealed differences that favored the use of mouth-to-mouth over a pocket mask in terms of the tidal volume [22]. The present study cannot conclude that one method is better than the other, as both achieve good-quality resuscitation. Thus, both ventilation systems, when used with the OTH technique, are viable and effective in situations with limited human and health resources [14,17].

Another relevant finding pertains to CPR quality. Although the present study presents a simulation and differs greatly from real-life conditions, a pre-established simulation quality criterion for CPR requires that a 70% success rate be achieved or surpassed when performing resuscitation maneuvers [19]. In the present study, this benchmark was greatly surpassed, particularly in terms of the chest compressions where the median reached 100% in depth, rate, and recoil. Prior to the assessment, the participants completed a training session using visual feedback devices, which likely contributed to their performance. In addition, the fact that the participants were all sports science students—a profile typically associated with good physical condition, high motor skill proficiency, and strong learning capacity—may also help to explain the high quality of CPR observed. While chest compressions are generally easier than ventilation, the quality of ventilations remains crucial and most were performed effectively in the present study. This is especially important when it comes to treating cardiac arrest due to drowning (asphyxia etiology) [7], where early oxygenation can facilitate the return of spontaneous circulation [23].

For all of the participants, the present study represents the first time that they had ever handled equipment. Despite this, their performance was similar to or better than that seen in other more experienced study populations, such as basic life support instructors [15] and lifeguards [14], and clearly superior to less-trained personnel such as an airline cabin crew, who were unable to successfully ventilate with a mouth-to-pocket mask [24]. Thus, introductory training and a prior skills assessment (as conducted in the present pilot study) can provide valuable feedback to first responders and help them to be able to decide whether to ventilate and how to do so effectively.

### 4.1. Study Limitations

The present pilot study has a number of limitations that should be considered. The first pertains to the sample selection and the study’s small sample size, which might prevent the findings from being extrapolated to the entire population trained in these types of ventilation techniques. Additionally, particular caution should be exercised when extrapolating the present findings to other rescuer profiles, as the participants were young lifeguards who were university students on a sports science program, who are typically characterized by good physical fitness, mobility, and good learning capacity and manual skills. Thus, caution is needed before extrapolating the findings to other populations. The second limitation is that the data were gathered from a simulation setting using a resuscitation manikin. This allows for consistent experimental conditions to evaluate response to cardiac arrest; however, it does not account for the characteristics of a real victim (size, chest wall compliance, ease of sealing the mask with the face, etc.). Finally, differences were only compared between two ventilation techniques (BVM and pocket mask) in the same OTH position. This prevents the findings from being extrapolated to other positions (standard or straddle) or other types of ventilation methods (e.g., mouth-to-mouth).

### 4.2. Implications for Practice and Future Research Perspectives

The present pilot study suggests that a single rescuer may be able to perform OTH CPR with a mouth-to-pocket mask as effectively as with a BVM. The implementation of these OTH techniques could provide an especially useful alternative in scenarios where the rescuer is expected to act alone, particularly in special circumstances such as confined spaces, whilst also ensuring high-quality CPR. It is also important that OTH techniques (including different ventilation methods) are taught as a part of basic life support programs for first responders (e.g., lifeguards). A time investment of just 10 min in ventilation with each technique (whilst also ensuring quality: a certified instructor and four manikins connected to a CPR feedback system for every eight students) appears to be sufficient for quality CPR provision. BVM remains the preferred option for professional rescuers due to biosafety and oxygenation considerations. It avoids direct contact with the victim’s airway and provides higher oxygen concentrations than the exhaled air delivered through a pocket mask. For individuals trained in CPR, supporting pocket mask use when BVM is unavailable would be beneficial. To achieve this, it is necessary to incorporate pocket mask training into basic life support training and promote and facilitate its accessibility as personal protective equipment for first responders and in first aid kits, including those attached to AEDs.

In 2015, OTH resuscitation was proposed as a good alternative when standard resuscitation cannot be performed [25]. However, in light of the present findings and those highlighted by other related research, it may be worth considering whether OTH (regardless of available ventilation equipment) should be promoted as the ‘first option’ for trained rescuers who feel comfortable with this technique.

Future studies should aim to evaluate the effectiveness of CPR using the OTH technique in various clinical settings and with different rescuer profiles, including those with less experience and different levels of physical fitness. It may also be of interest to examine the influence of factors such as rescuer fatigue or prolonged resuscitation duration on CPR quality. Finally, future studies should include real-life resuscitation scenarios to allow for a realistic assessment of single first responder use of the different types of ventilation during application of this technique and subsequent outcomes.

## 5. Conclusions

For a single trained first responder, quality resuscitation while positioned OTH and performing mouth-to-pocket mask ventilation is feasible in the same way as OTH with a BVM. Resuscitation quality was optimal regardless of the method applied. The OTH technique with a pocket mask may represent a feasible alternative in scenarios in which only a single rescuer is present and this device is available. This approach could be particularly useful in special resuscitation circumstances in which the standard position is not viable, or in which the first responder prefers this technique over the standard technique, counts on adequate training and sufficient experience, and considers this technique to be suitable.

## Figures and Tables

**Table 1:** CPR variables.

	BVM-OTH		Pocket Mask-OTH		Difference	*p*-Value
CPR Performance	Median	IQR	Median	IQR		
CPR quality (%)	98	(97–99)	99	(94–99)	0.0 (−1.0–4.0)	0.31
Ventilations						
Number of effective initial ventilations	5	(5–5)	5	(5–5)	0.0 (0.0–0.0)	0.06
Ventilation quality (%)	100	(95–100)	99	(77–100)	0.0 (0.0–12.0)	0.05
Effective ventilations	9	(8–10)	10	(8–10)	0.0 (−1.0–1.0)	0.73
CC						
Quality of CCs (%)	99	(99–100)	99	(99–100)	0.0 (0.0–0.5)	0.24
Number of CCs	150	(149–152)	157	(150–165)	−9.0 (−11.0–−4.0)	<0.001
CC fraction (%)	69	(66–70)	72	(69–75)	−2.5 (−6.5–−1.0)	0.001
Average CC rate (CC/min)	111	(108–114)	111	(109–113)	−0.8 (−2.5–0.8)	0.31
CCs with correct rate (%)	100	(98–100)	100	(95–100)	0.0 (0.0–1.0)	0.50
CCs with correct depth (%)	100	(100–100)	100	(100–100)	0.0 (0.0–0.0)	0.26
CCs with correct recoil (%)	100	(100–100)	100	(100–100)	0.0 (0.0–0.0)	0.50

Note: BVM: Bag-valve mask. OTH: Over-the-head resuscitation. CPR: Cardiopulmonary resuscitation. IQR: Interquartile range. CC: Chest compression.

**Table 2:** Perceptions of the ease/difficulty of technique performance.

	BVM-OTH		Pocket Mask-OTH		Difference	*p*-Value
Perception of Ease/Difficulty of Performing CPR	Median	IQR	Median	IQR		
CPR (0–5)	1.5	(1.0–2.0)	2.0	(1.0–2.0)	0.0 (0.0–0.0)	0.82
Ventilations (0–5)	2.0	(1.0–3.0)	2.0	(1.0–2.3)	0.0 (0.0–1.0)	0.77
CC (0–5)	1.0	(1.0–2.0)	1.5	(1.0–2.0)	0.0 (0.0–0.0)	0.78

Note: BVM: Bag-valve mask. OTH: Over-the-head resuscitation. CPR: Cardiopulmonary resuscitation. IQR: Interquartile range. CC: Chest compression. Scale: (0: very easy; 1: easy; 2: somewhat easy; 3: somewhat difficult; 4: difficult; 5: very difficult).

## Data Availability

We declare that the data are not available due to privacy reasons.

## Abbreviations

The following abbreviations are used in this manuscript:

OTH	Over-the-head resuscitation technique
CPR	Cardiopulmonary resuscitation
CC	Chest compressions
BVM	Chest compressions
ERCGR	European Resuscitation Council Guidelines for Resuscitation

## References

[1] Zulhan M.I., Kumboyono K., Lestari R. (2024). Improving Cardiopulmonary Resuscitation Skills for Layperson in Cases of Heart Attack: A Scoping Review. Healthc. Low-Resour. Settings.

[2] Hwang C.W., Murphy T.W., Antoine J., Avery K.L., Carr C., Han F., Snipes G., Zhou S., Becker T.K. (2023). Cardiac Arrest: An Interdisciplinary Scoping Review of Clinical Literature from 2021. Front. Emerg. Med..

[3] Gräsner J.T., Wnent J., Herlitz J., Perkins G.D., Lefering R., Tjelmeland I., Koster R.W., Masterson S., Rossell-Ortiz F., Maurer H. (2020). Survival after Out-of-Hospital Cardiac Arrest in Europe—Results of the EuReCa TWO Study. Resuscitation.

[4] Cimpoesu D., Corlade-Andrei M., Popa T.O., Grigorasi G., Bouros C., Rotaru L., Nedelea P.L. (2019). Cardiac Arrest in Special Circumstances—Recent Advances in Resuscitation. Am. J. Ther..

[5] Slomine B.S., Nadkarni V.M., Christensen J.R., Silverstein F.S., Telford R., Topjian A., Koch J.D., Sweney J., Fink E.L., Mathur M. (2017). Pediatric Cardiac Arrest Due to Drowning and Other Respiratory Etiologies: Neurobehavioral Outcomes in Initially Comatose Children. Resuscitation.

[6] Van Beeck E.F., Branche C.M., Szpilman D., Modell J.H., Bierens J.J.L.M. (2005). A New Definition of Drowning: Towards Documentation and Prevention of a Global Public Health Problem. Bull. World Health Organ..

[7] Bierens J., Bray J., Abelairas-Gomez C., Barcala-Furelos R., Beerman S., Claesson A., Dunne C., Fukuda T., Jayashree M., T Lagina A. (2023). A Systematic Review of Interventions for Resuscitation Following Drowning. Resusc. Plus.

[8] Truhlář A., Deakin C.D., Soar J., Khalifa G.E.A., Alfonzo A., Bierens J.J.L.M., Brattebø G., Brugger H., Dunning J., Hunyadi-Antičević S. (2015). European Resuscitation Council Guidelines for Resuscitation 2015: Section 4: Cardiac Arrest in Special Circumstances. Resuscitation.

[9] Szpilman D., Bierens J.J.L.M., Handley A.J., Orlowski J.P. (2012). Drowning. N. Engl. J. Med..

[10] Dezfulian C., McCallin T.E., Bierens J., Dunne C.L., Idris A.H., Kiragu A., Mahgoub M., Shenoi R.P., Szpilman D., Terry M. (2024). 2024 American Heart Association and American Academy of Pediatrics Focused Update on Special Circumstances: Resuscitation Following Drowning: An Update to the American Heart Association Guidelines for Cardiopulmonary Resuscitation and Emergency Cardiovascular Care. Circulation.

[11] Lott C., Truhlář A., Alfonzo A., Barelli A., González-Salvado V., Hinkelbein J., Nolan J.P., Paal P., Perkins G.D., Thies K.C. (2021). European Resuscitation Council Guidelines 2021: Cardiac Arrest in Special Circumstances. Resuscitation.

[12] Szpilman D., Webber J., Quan L., Bierens J., Morizot-Leite L., Langendorfer S.J., Beerman S., Løfgren B. (2014). Creating a Drowning Chain of Survival. Resuscitation.

[13] Adelborg K., Bjørnshave K., Mortensen M.B., Espeseth E., Wolff A., Løfgren B. (2014). A Randomised Crossover Comparison of Mouth-to-Face-Shield Ventilation and Mouth-to-Pocket-Mask Ventilation by Surf Lifeguards in a Manikin. Anaesthesia.

[14] Barcala-Furelos R., Carracedo-Rodríguez E., Lorenzo-Martínez M., Alonso-Calvete A., Otero-Agra M., Jorge-Soto C. (2023). Assessment of Over-the-Head Resuscitation Method in an Inflatable Rescue Boat Sailing at Full Speed. A non-inferiority pilot study. Am. J. Emerg. Med..

[15] Perkins G.D., Stephenson B.T.F., Smith C.M., Gao F. (2004). A Comparison between Over-the-Head and Standard Cardiopulmonary Resuscitation. Resuscitation.

[16] Ćwiertnia M., Kawecki M., Ilczak T., Mikulska M., Dutka M., Bobiński R. (2019). Comparison of Standard and Over-the-Head Method of Chest Compressions during Cardiopulmonary Resuscitation—A Simulation Study. BMC Emerg. Med..

[17] Nasiri E., Nasiri R. (2014). A Comparison between Over-the-Head and Lateral Cardiopulmonary Resuscitation with a Single Rescuer by Bag-Valve Mask. Saudi J. Anaesth..

[18] Olasveengen T.M., Semeraro F., Ristagno G., Castren M., Handley A., Kuzovlev A., Monsieurs K.G., Raffay V., Smyth M., Soar J. (2021). European Resuscitation Council Guidelines 2021: Basic Life Support. Resuscitation.

[19] Perkins G.D., Colquhoun M., Simons R., Colquhoun M., Handley A., Evans T. (2004). Training manikins. ABC of Resucitation.

[20] Gaszyński T., Borkowski B., Przybyt-Sibelska K., Chmiela K. (2021). A Comparison of Mouth-to-Mouth, Mouth-to-Pocket Face Mask and Bag Valve Mask Ventilation during Lifeguards’ CPR: A Manikin Study. Emerg. Med. Serv..

[21] Gerber L., Botha M., Laher A.E. (2021). Modified Two-Rescuer CPR With a Two-Handed Mask-Face Seal Technique Is Superior To Conventional Two-Rescuer CPR With a One-Handed Mask-Face Seal Technique. J. Emerg. Med..

[22] Adelborg K., Dalgas C., Grove E.L., Jørgensen C., Al-Mashhadi R.H., Løfgren B. (2011). Mouth-to-Mouth Ventilation Is Superior to Mouth-to-Pocket Mask and Bag-Valve-Mask Ventilation during Lifeguard CPR: A Randomized Study. Resuscitation.

[23] Szpilman D., Soares M. (2004). In-Water Resuscitation—Is It Worthwhile?. Resuscitation.

[24] Handley A.J., Handley J.A. (2004). Performing Chest Compressions in a Confined Space. Resuscitation.

[25] Monsieurs K.G., Nolan J.P., Bossaert L.L., Greif R., Maconochie I.K., Nikolaou N.I., Perkins G.D., Soar J., Truhlář A., Wyllie J. (2015). European Resuscitation Council Guidelines for Resuscitation 2015: Section 1: Executive Summary. Resuscitation.

